# Understanding the public voices and researchers speaking into the 5G narrative

**DOI:** 10.3389/fpubh.2023.1339513

**Published:** 2024-01-12

**Authors:** Steven Weller, Julie E. McCredden

**Affiliations:** ^1^Centre for Environmental and Population Health, School of Medicine and Dentistry, Griffith University, Brisbane, QLD, Australia; ^2^Oceania Radiofrequency Scientific Advisory Association Inc. (ORSAA), Scarborough, QLD, Australia

**Keywords:** 5G narrative, wireless radiation, environmental health, health advocacy, risk management, precautionary approach, conflicts of interest, science communication

## Abstract

The many different voices speaking into the current narrative surrounding the health effects of 5G technologies necessitate an exploration of the background of the various published author-spokespersons and their potential motives. This has been attempted recently by de Vocht and Albers. However, that opinion piece used a narrow investigative lens, resulting in an undermining of both the rationality of the concerned general public and the motives of specific researchers. At the same time, biases, conflicts of interest, and flaws found in “independent” reviews were not considered. To address these oversights, an evidence-based appraisal of public opinion and the scientific caliber of authors involved in the 5G health discussion is warranted. Subsequently, this review article presents an analysis of the available Australian data representing public voices, while also conducting a broader investigation of the level of expertise of recent author-spokespersons based on their experience as scientists, particularly in the area of health effects of radiofrequency electromagnetic fields. This review thus attempts to more clearly illustrate for the reader the caliber and motives of the voices speaking into the 5G narrative. The article concludes with a set of questions that need to be answered to enable scientists to advise policy makers more effectively on matters of 5G and public health.

## 1 Introduction

The rollout of 5G communications technology worldwide has been welcomed by industry and governments with repeated reassurances of safety provided to the public (1, 2). On the other hand, members of the public have aired concerns about health, privacy and security issues (3). At the same time, several authors have published articles calling for a moratorium based on existing evidence regarding health risks from electromagnetic fields, ranging from low frequency fields through to extremely high frequency microwaves [collectively denoted here as radiofrequency electromagnetic fields (RF-EMF)]. Some authors have criticized governments for allowing an unfettered rollout of 5G, by prioritizing economic interests over and above the wellbeing of the public (4). Due to these noticeable differences in opinion, it is therefore imperative to investigate those researchers whose voices are contributing to the 5G health and safety narrative, in terms of their background and qualifications, while also comparing these attributes with the stance they have taken. It is also important to understand the basis and validity of opinions emanating from the general public as part of their contributions to the narrative.

## 2 Investigation

### 2.1 Understanding public voices

Public concerns have been described in a recent opinion piece (5) as representing only a small “pocket” of society whose opinions originate from beliefs in conspiracy theories or mere “perceptions” of health risks. Understandably, public awareness of the potential risks associated with radiofrequency exposure is limited to those who have taken the time to investigate the problem for themselves, because there is very little to no information being disseminated by industry or government regulatory bodies around the world. Nevertheless, so as to examine the claims made by the aforementioned opinion piece, two documented sources of public opinion expressed in Australia in 2019–2020 were investigated.

#### 2.1.1 Amount of public concern

The majority of the public have given support to 5G technology, as indicated in a 2019 Roy Morgan survey of 626 Australians (6). However, in that survey, close to one quarter of the respondents (26%) acknowledged concerns about health risks while one fifth (20%) acknowledged concerns about security. Furthermore, during the Australian 2019–2020 parliamentary inquiry into 5G technology (7), at least three towns and large suburbs were in the process of taking legal action to prevent small cell deployments. This level of public concern is more than a mere “pocket.”

#### 2.1.2 Rationality of concerns

Analysis of the 2019 Australian 5G parliamentary inquiry shows that public concerns over rising levels of exposure were based on facts using published government reports; i.e., Electro Magnetic Energy (EME) reports suggesting that proposed 5G upgrades to existing base stations would raise background levels by up to 1,800% or more (8). Therefore, members of the public were not creating imaginary “perceptions” on which to base their concerns.

#### 2.1.3 Size of rational vs. conspiracy theory groups

A thematic and quantitative analysis of data from 531 submissions to the Australian 5G inquiry (3) also provides some insight into the main concerns expressed by various stakeholder groups (9). The major themes of concern expressed by members of the pubic were as follows: health and safety risks (89%), increasing radiation levels (51%), environmental risks (44%), and that the 5G rollout is a non-consensual experiment on the public and environment (24%). Some claimed that 5G is based on military technology (11%; which is factual because 5G phased array and beam steering capabilities originate from military radar technology) (10, 11).

Concerns about misinformation or misunderstandings were expressed on both sides of the debate (~5%); i.e., industry expressed concern about public misunderstanding (0.7% i.e., four submissions) and members of the public (4% i.e., 24 submissions) expressed concern about misinformation from industry or the Australian Radiation Protection and Nuclear Safety Agency (ARPANSA). Unsubstantiated claims were made by a small minority of both sides of the debate. There were public concerns that 5G has been developed as a military weapon or for crowd control, while one Telco referenced a New York Times article suggesting that those who are concerned about 5G technology are being influenced by “Russian interests” so as to hold the West back. Altogether, the unsubstantiated claims made up 2% of the public submissions. This is a small proportion that cannot be deemed to be a “compounding factor” on the rest of the public concerns as suggested by de Vocht and Albers (5).

Overall, the majority of public submissions constituted rational and informed voices objecting to the 5G rollout. Many of these submissions (41%) provided multiple references to peer reviewed research to support their statements. Most (90%) of the public submissions expressed concerns that can be grouped into two major themes: (i) potential risks to health and security and (ii) absence of risk management. These two issues are the remit of governments based on the acts of parliament under which they operate. Given the current lack of clear understanding in the published literature regarding the long-term effects of microwave exposures on human and environmental health (12, 13), the public rightfully require their leaders and public servants to develop an adequate risk management policy on the issue of RF-EMF exposures and health.

### 2.2 Classifying research authors

Past analyses of papers in the field of RF-EMF and health have shown that industry funded papers (as declared by authors) are less likely to report significant findings (14, 15). However, the opinion piece by de Vocht and Albers (5) used a different classification scheme for authorship, with confusing results. Researchers aligned with industry were labeled as “independent,” while reputable industry-independent scientists who speak against 5G were categorized under “activism” or labeled as “white hats.” This labeling seems to be skewed and missing critical analysis of the important issues, as described below.

#### 2.2.1 Authorities not activists

To use the label “activism” to describe scientists who speak out on a particular issue without fully clarifying the meaning can demonstrate potential bias. The Council of Europe recognizes activism as an important component of civil society, ensuring protection of human rights and the environment (16). However, when the term “activist” is applied to scientists who speak out on a particular issue, the negative media slant on this term can give an inaccurate impression of extremist or radical behavior. Furthermore, “activist” is not precise enough. A more accurate term for a highly experienced scientist who has deep subject matter expertise, recognized by peers and therefore is able to speak with precision and breadth in their area of expertise, is an “authority.” Government policy makers or the private sector, may ask an authority to provide opinion in their area of expertise. In response, an authority applies the facts of their discipline to the matter at hand, using knowledge and skills that an activist may not possess. When an authority sees the need to speak out on an issue unsolicited, they are undertaking “advocacy” work, which is an expected role for professional scientists to play when required (e.g., Einstein was a pacifist who advocated for a ban on nuclear testing).

### 2.3 Researcher experience relevant to RF-EMF

So as to give a full account of the real level of expertise and the caliber of authors who have published within the 5G narrative, it is instructive to look at their publication history and research experience. This provides a more well-rounded analysis than critiquing each author on the basis of a recent paper, as was performed by de Vocht and Albers (5). A fuller appraisal of each author will assist the reader to decide how much weight and attention they should pay to the opinions that each has contributed toward the 5G narrative.

A detailed analysis of the background of each recent author was therefore conducted by investigating each according to (i) whether they come from a clinical, radiological or scientific research background; (ii) how much experience they have in their own field; (iii) the type of research work they have conducted investigating RF-EMF exposure and health (a scientist with first-hand experimental experience in biological or health effects, a theoretical physicist/engineer who conducts modeling, a researcher who uses the works of others to build new theory or methodologies, or an analyst who investigates the work of others and makes reasoned arguments from this analysis) and (iv) their level of experience in the field of RF-EMF and health (highly experienced, experienced or novice) based on the number of papers published and the number of years involved.

We based this investigation on the list of first authors presented by de Vocht and Albers (5). However, we also included other important first authors of review papers or reports that were missing from that analysis, or who have published since August, 2021. These authors are: (i) Dr. James Lin, a highly experienced radiological engineer and previous International Commission on Non-Ionizing Radiation Protection (ICNIRP) member, who has published two recent papers (13, 17) that question the safety of 5G and challenge the underlying scientific basis of the updated 2020 ICNIRP guidelines; (ii) Professor Igor Belyaev, a giant in the field of experimental biophysics, who has recently published two papers on health risks from exposure to wireless signals and the invalid health assumptions underlying the FCC and ICNIRP exposure limits (18, 19); and (iii) Professor Fiorella Belpoggi who has conducted experimental work investigating cancer and toxicology in animals for over two decades, and was previously the director of research at the Italian Ramazzini Institute where evidence for cancer due to whole body RF-EMF exposures in rats was found. Belpoggi also wrote the 2020 European Parliament commissioned report (20) on the health effects of 5G, reviewing papers since 2016.

Our background search of the full list of authors (see Supplementary material 1) revealed that authors who have been warning of biological and health effects range from experienced doctors and analysts through to highly qualified experts. That is, while some are highly experienced in their own fields of health or public policy, they have only in the last few years been applying their expertise to the field of RF-EMF and health. Thus, they are classified as analysist's or researchers but not experts. For example, Di Ciaula is a highly experienced medical researcher in the area of gallbladder disease, who has recently applied his medical understanding so as to conduct a systematic review of the biological effects of millimeter waves (following the PRISMA guidelines closely). Di Ciaula is thus classified as an analyst rather than as an experienced RF-EMF researcher. Similarly, Frank, a highly experienced epidemiologist in his own field, has recently written a critique of the advice regarding 5G being given to policy makers by government and non-government agencies. Therefore, Frank is also classified as an RF-EMF analyst.

There are several authors warning of biological and health effects who are highly experienced in the area of RF-EMF and health (i.e., Belyaev, Belpoggi, Hardell, Lin, and Miller). These authors are bona fide authorities with two or more decades of experimental or epidemiological research experience, and hundreds of publications covering RF-EMF and health. Belyaev, Hardell, and Miller were all members of the previous World Health Organization (WHO) International Agency for Research on Cancer (IARC) working group looking into the carcinogenic effects of RF-EMF, and Lin was a long-term member of ICNIRP. Prior to investigating RF-EMFs, Hardell investigated the links between other agents and cancer such as dioxins, PCBs and glyphosate, advocating for recognition of carcinogenicity, while government regulatory bodies and industry-linked scientists tried to discredit him. History seems to be repeating itself for Hardell today as he continues to advocate for recognition of the link between mobile phone use and brain tumors (21).

Overall, it is clear that these scientists are well-qualified to advocate for precaution regarding 5G because their opinions are based on discoveries that have arisen from their own rigorous research. As true experts they have earned the right to be heeded in their weighty statements of concern regarding health risks from RF-EMFs. It is inexplicable therefore, why two of these authorities, Hardell and Miller, were demoted by de Vocht et al. (5) as “white hats” and classified under “activism.” Such skewed labeling introduces doubt regarding the motives of sound scientists and detracts from their credentials as experts in this field. This unscrupulous “discredit the scientist” strategy has been used previously by the tobacco industry so as to demote the research of scientists presenting results showing health risks from smoking (22).

#### 2.3.1 Membership of professional associations

All of the above researchers are affiliated with professional advocacy organizations, which should not be misconstrued as a conflict of interest, as suggested by de Vocht and Albers (5), but instead, regarded as part of their ethical obligations. For example, the International Radiation Protection Agency (IRPA, the professional umbrella organization sitting over ICNIRP) was established by researchers and technicians in 1965, so as to promote health issues arising within radiation physics (23). These efforts eventually led to non-ionizing radiation protection becoming a priority in government policy internationally, with subsequent industry regulations. Similarly, concerned scientists and medical experts around the world today are forming organizations to bring an awareness of health issues related to non-ionizing radiation, and the inadequacy of the current international ICNIRP guidelines (24) to protect the general public. The International Commission on the Biological Effects of Electromagnetic Fields (ICBE-EMF, an international expert NGO alternative to ICNIRP) has been established recently so as to bring to policy makers the awareness of health risks along with positive solutions toward mitigating the effects of RF-EMFs on health (18).

The full summaries and subsequent classifications of each author resulting from the above background search are presented in Supplementary material 1. In addition, Supplementary material 2 contains the abstracts of pertinent papers by each author extracted from the ODEB^1^ collection.

### 2.4 Hidden conflicts of interest need to be exposed

When background affiliations are being investigated, research projects linked with government or industry interests need to be noted. For example, the tobacco industry implemented strategies for suppressing evidence regarding health effects from smoking, which included: funding research that supported the industry position, setting up official “review” bodies who concluded that results were inconsistent, and funding central research centers that would supposedly be *a focal point organization of the highest caliber to sponsor and foster quality, objective research … to effectively communicate research findings to a broad scientific community* [(22), p. 202]. Similar patterns can be observed in the field of health effects of wireless radiation, as follows:

- Researchers working at university institutions with laboratories funded by, or in partnership with telecommunication industries seem unable to maintain both their jobs and their independence from these industries. For example, after Dr. Bruce Hocking, former Chief Medical Officer for the Australian telecommunications company Telstra, published on neurological changes in his patients from exposure to their mobile phones (25), he was made redundant. Similarly, after Fred Hollows found that Telstra linesmen exposed to microwaves were three times more likely to develop cataracts (26), Telstra complained to Hollows' university. Hollows received no further funding to conduct follow-up studies (27).
- The international advisory body, ICNIRP, has members with a history of industry affiliations (28). This creates an inherent industry-bias within ICNIRP members, which has been noted by the Ethical Council at the Karolinska Institute in Stockholm (29) and by the Court of Turin where evidence provided by ICNIRP was deemed biased and not reliable (30).
- Government advisory agencies are unable to make independent statements about health and exposures, because they are expected to support government plans for comprehensive internet of things (IoT) and smart cities, which are dependent on wireless technologies (31, 32). Moreover, Australia's advisory agency ARPANSA is not permitted to make changes to the RF standard to protect health and the environment if it would prejudice the departments of Defense or National Security (33). The need to support these two agencies most likely creates pressure on ARPANSA not to oppose further wireless technology rollouts or lower exposure limits to improve public safety.
- Members of government advisory agencies are also members or associates of industry-linked ICNIRP (34); e.g., Karipidis, an ARPANSA researcher, is also a member of ICNIRP.
- The supposedly independent international advisory body, the WHO International EMF project is strongly influenced by industry in the form of the International Telecommunications Union and ICNIRP [(35), p. 5–6].
- Government regulatory agencies and advisory bodies may derive their income from industry via RF spectrum sales. For example, in Australia in 2021, $700,000 p.a. of the funding for the government's advisory agency ARPANSA came from revenue from spectrum licenses collected by the self-regulated industry-friendly Australian Communications Media Authority (ACMA). Then, of the money received by ARPANSA that year, a portion went to the WHO EMF project and to ICNIRP (36). ARPANSA state publicly that they take advice from the WHO and from ICNIRP, as if these are independent bodies advising ARPANSA. However, ARPANSA supports the WHO EMF financially and contributes to the WHO EMF project reports (35). Overall, several of the same people appear in all three committees (WHO EMF, ICNIRP and ARPANSA) and money flows from one group to another. Minuted evidence of ARPANSA's strategy to maintain this interdependence between ARPANSA, ICNIRP, research institutions, and the WHO EMF project is given in Supplementary material 3.

Such conflicts of interest make it unclear whose interests are being represented when a member of one these groups speaks into the 5G narrative. For example, when a WHO webpage on potential health risks from 5G informs that *Provided that the overall exposure remains below international guidelines, no consequences for public health are anticipated* (37), it is uncertain who is really speaking: ARPANSA, ICNIRP, ACMA,^2^ or the telecommunications industry. Similar inter-relationships between government agencies, industry, ICNIRP, and WHO have been documented for other countries (29, 34). It appears that prestigious and supposedly independent health bodies along with the regulatory agencies advising on the 5G rollout have *significant elements in that apparatus* [that] *appear to have been captured by vested interests* [(38), p. 565].

So as to discern real from illusory independence therefore, clear categories of “Government/ICNIRP” and “Institution/industry” need to be available for classifying the affiliations of the author-spokespersons within the 5G narrative. When this system is used (see Supplementary material 1), we find that two of the researchers deemed to be “independent” in the opinion article by de Vocht and Albers (5) need to be reclassified, due to Telstra funding of Wood's laboratories (39, 40) (Institution/industry), and Karipidis' employment by ARPANSA and membership of ICNIRP (41) (Government/ICNIRP).

#### 2.4.1 Hidden industry influences via co-authorship

It is also instructive to look at the coauthors of each of the main author-spokespersons. Supplementary material 4 lists the authors and co-authors and their links with industry. Wood, a coauthor of Karipidis, has published with several authors closely linked to industry. Kenneth Foster has co-authored with Bushberg, Simkó, and Wood, and has several papers funded by the wireless industry. Foster was mentored by the ex-German biophysicist Herman Schwan, who modeled effects using macro biophysics, but did not incorporate the developing biological or quantum perspectives and was thus unable to let go of his “thermal only” position (see Supplementary material 1). Schwan's position along with industry interests are still influencing today's 5G narrative, via the influence of Foster as co-author on many publications.

### 2.5 Reclassifying the voices of the 5G narrative

The authors speaking into the 5G narrative since 2018 have been classified using the above dimensions. Table 1 shows that these authors can be grouped into those who take a precautionary and risk assessment approach and those who do not.

**Table 1:** Classification of recent 5G narrative authors according to experience and approach.

**Publication date of 5G article**	**First author**	**Qualification**	**Publication type**	**Expertise in microwave research and health**	**Funding or associations**	**Effects found in past EMF research**	**Identifies health risk**	**Risk assessment approach**
**Author advises precaution in one or more publications**			
Feb 2018 (42)	Di Ciaula	Medical Doctor	Systematic-style review	Analyst	Independent Health Advocate	N/A	Yes	Yes
Apr 2018 (43)	Russell	Medical Doctor	Narrative review	Experienced Analyst	Independent Health Advocate	N/A	Yes	Yes
Aug 2018 (44)	McClelland	Medical Doctor	Commentary	Experienced Medical Practitioner	Independent	N/A	Yes	Yes
Aug 2019 (45)	Miller	Medical Doctor	Narrative review	Highly Experienced Epidemiologist (in low frequency EMFs)	Independent Health Advocate	Yes	Yes	Yes
Oct 2019 (19)	Belyaev	Ph.D. Radiobiology (biophysics), Doctor of Science Genetics	Narrative review	Highly Experienced Biophysicist	Independent	Yes	Yes	Yes
Jan 2020 (46)	Hardell	Medical Doctor, Oncologist, Epidemiologist	Commentary	Highly Experienced Oncologist and Epidemiologist	Independent Health Advocate	Yes	Yes	Yes
Jul 2020 (29)																
Jun 2021 (47)																
Jan 2023 (48)																
Jan 2020 (49)	Kostoff	Ph.D. Public Policy	Narrative review	Experienced Analyst	Independent Health Advocate	N/A	Yes	Yes
Mar 2020 (20)	Belpoggi	Ph.D. Oncology, toxicology	European Parliament Commissioned Report review style	Highly Experienced Oncologist and Toxicologist	Independent Health Advocate	Yes	Yes	Yes
May 2020 (50)	Nizhelska	Ph.D. Biophysics	Narrative review	Experienced Biophysicist	Government (Ukraine)	Yes	Yes	Yes
Aug 2020 (12)	Leszczynski	Ph.D. Biochemistry/Cell biology	Systematic-style review	Highly Experienced Biological Scientist	Past: Government/Institution; Present: Independent	Yes	Too Early Insufficient Research	Partial
Jan 2021 (38)	Frank	Medical Doctor/Epidemiologist	Essay	Analyst	Independent Health Advocate	N/A	Yes	Yes
Sep 2020 (51)	Lin	Ph.D. Electrical Engineering	Commentary	Highly Experienced Radiological Engineer	IEEE Member, Past: Military, ICNIRP Member; Present: Independent	Yes	Yes	Yes
Jan 2022 (13)																
Feb 2023 (52)																
**Author does not advise precaution**			
Sep 2019 (53)	Simkó	Ph.D. Cell Biology	Systematic-style review	Highly experienced ELF Biophysicist	Industry (Deutsche Telekom Technik)/SCENIHR	Yes	No	No
Jun 2020 (54)	Bushberg	Ph.D. Radiological Physics	Narrative review	Highly Experienced Radiological Physicist	IEEE member; Industry	No	No	No
Mar 2021 (55)	Jargin	Medical Doctor/Pathologist	Letter to the editor	Novice Commentator	Independent	N/A	No	No
Mar 2021 (56)	Karipidis	Physicist; Ph.D. Epidemiology	Systematic-style review	Analyst	Government (ARPANSA)/ICNIRP	No	No	No
Mar 2021 (57)	Wood	Ph.D. Theoretical Biophysics	Meta-analysis	Highly experienced in RF-EMF theory and modeling	Industry/Institution partnership (Telstra)	Yes	No	No

#### 2.5.1 Expertise, industry links, and precautionary position

Authors that recommend a precautionary approach and apply a risk assessment philosophy are predominantly medical experts, epidemiologists, biological scientists and biophysicists, or analysts whose papers conclude that there is evidence for risk of harm. Conversely, authors who do not advise precaution or apply a risk assessment approach for evaluating potential hazards are typically connected to industry or ICNIRP and have more expertise in physics or engineering than in biology or biophysics. While two of these authors (Bushberg and Wood) are highly experienced physicists, their expertise is in dosimetry or theoretical modeling and not health. These “no precautionary approach” authors are not experts in RF-EMF and health and are all linked with industry, either directly or indirectly via their co-authors. One anomaly within this pattern is Simkó, a highly experienced EMF and health scientist funded by industry, who finds no confirmed evidence of harm, but calls for a risk policy to be developed by governments (58).

#### 2.5.2 Risk management and precautionary approach neglected

Given that the stakes are very high, the public rightfully require their leaders and public servants to develop an adequate risk management policy on the issue of RF-EMF exposures and health (59). Unfortunately however, as current members of IRPA who are steeped in radiation protection philosophy have noted, there is currently an absence of radiation hygiene being applied by ICNIRP, resulting in a lack of precautionary advice being provided to governments internationally (60). Many ICNIRP-aligned government and industry-linked organizations do not take a risk management approach and there is a reluctance to discuss any potential risks. For example, precautionary text has been removed from the updated ARPANSA RF Protection Standard (RPS S-1) (61). A serious consequence of such a laissez-faire approach to risk management is that there has been no testing for biological/health effects of RF-EMF exposures under real-world use conditions, or any post-market surveillance looking at health outcomes from long term chronic exposures. If you don't look, you won't find. This allows industry, ICNIRP and government regulators to claim, “there is no established evidence that RF-EMF exposure below ICNIRP limits is harmful.”

The majority of the non-precautionary approach authors summarized in Table 1 are either members of ICNIRP and ARPANSA or coauthors on papers with members of industry-linked advisory bodies.

### 2.6 Biases in reviews

Reviews or systematic reviews being authored by researchers with potential conflicts of interest may contain biases, and therefore may not be the gold standard reviews that are anticipated:

*Factors such as the selection and inclusion criteria, the search strategy, the use of multiple reviewers, and the assessment of study quality can impact the reliability of the review. Also, the expertise and the opinions of the scientific team preparing systematic review's protocol and performing the review might introduce quality bias and bias of own opinions into the process* (62).

As an example, both Karipidis and Simkó reviews reported evidence for effects within the body of their papers: i.e., Eighty *percent of the in vivo studies showed responses to exposure, while 58% of the in vitro studies demonstrated effects. The responses affected all biological endpoints studied* [(53), p. 3,406]. However, Simkó et al. (who received funding from Deutsche Telekom Technik) gave a muted conclusion, merely suggesting that more research is needed, and Karipidis (with links to industry as shown above and in Supplementary material) concluded that there is no confirmed evidence of harm and health risks were not discussed. A published critique (63) of the Karipidis et al. review of 5G and health effects (56) [and by association, Wood's meta-analysis (57) because it used the same underlying data] describes in great detail how these papers suffered from multiple flaws, biases and inconsistent logic that invalidated findings and conclusions.

### 2.7 Watering down positive results

When the data in any study is being analyzed and communicated, it is important to distinguish “fact” (raw data) from “inference” (statements derived from data using scientific reasoning) and concluding opinions (that translate the results and analysis into real world implications). It is the final opinions that are the takeaways for the reader or the policy maker. Researchers can publish or publicly state opinions that construe their factual results as having less importance than the context may indeed warrant.

When conducting reviews, industry-linked researchers can also water down results. This is achieved by rejecting studies that show positive effects on the basis of the reviewers' opinion regarding supposed “methodological flaws” that are not clearly defined (64). Furthermore, unusual or unexpected results are dismissed as “inconsistent,” “not replicated,” or “possible artifacts of the testing process” rather than following them as possible leads for further investigation as is normally expected in research. Moreover, great emphasis is placed on inadequate dosimetry in otherwise sound papers, thus discrediting papers with positive results. Focusing on measurement accuracy of exposure levels rather than health effects was a strategy used by the tobacco industry (22).

### 2.8 Changing vs. unchanging conclusions

When anomalous findings arise, scientists who are grounded in their data are expected to subsequently change their frameworks or directions. The historical content of the publications of scientists who recommend a precautionary approach reveals how they have been transparent about past inconsistencies in results. However, over time, based on converging results from their own data and that of others, these authors have become more convinced that there are serious biological or health related effects. On the other hand, inspection of both the primary and review papers of industry linked researchers reveals that most have not changed their conclusions for several decades (see Table 2).

**Table 2:** Opinions of authors over time regarding health effects linked to RF-EMF exposures (Pertinent phrases are in bold - our emphasis).

**Author**	**Early conclusions/statements**	**Recent conclusions/statements**
Belyaev	…at present **there is no experimental evidence for the mutagenicity of millimeter waves at non-thermal level**. At first sight, with the presence of epigenetic effects of resonant EHF EMR, this seems to be paradoxical [(65), p. 17].	MMW inhibited repair of DNA damage induced by ionizing radiation at specific frequencies and polarizations. To what extend the 5G technology and the Internet of Things will affect the biota and human health is definitely not known. However, based on possible fundamental role of MMW in regulation of homeostasis and almost complete absence of MMW in atmosphere … **the health effects of chronic MMW exposures may be more significant than for any other frequency range** [(19), p. 111].
Hardell	Somewhat increased risks were found for amateur radio operators (OR 2.2; CI 0.7–6.6), work with radar equipment (OR 2.0; CI 0.3–14.2) and engineers in electronics and telecommunication industry (OR 2.3; CI 0.8–6.7) based on few exposed subjects, however. Video display unit work gave OR 1.5; CI 0.98–2.3 and for exposure 480 working days (median number) the risk increased further to OR 1.8; CI 1.1–3.2. Because of low numbers of exposed subjects in some calculations **some of these results might be spurious and need to be further studied** [(66), p. 1,299].	These now presented **symptoms of the microwave syndrome were caused by non-thermal effects from RF radiation** and highlight that **the ICNIRP guidelines used in most countries including Sweden do not protect human health**. Guidelines based on all biological negative effects from RF radiation are urgently needed, as well as monitoring human health, not the least due to rapidly increasing levels of exposure [(48), p. 1,112].
Wood	… The average frequency from Fourier spectra of these periods showed significant alteration in one category only: PW [Pulsed Wave] exposure of activated cells. Conclusions: Th**ere is no clear indication that RF emissions from mobile phones are associated with any changes** in calcium levels or calcium signaling in lymphocytes [(67), p. 1,207]. The results suggest that MP exposure may affect neural activity, particularly in proximity to the phone, **however caution should be applied due to the small sample size** [(68), p. 171].	**Overall, the results of this study do not confirm an association between low-level MMWs and biological effects** [(57), p. 606].
Foster	**The limited number of technology-specific bioeffects studies done to date are very mixed in terms of quality and outcome**. Unequivocally, the RF exposures from Wi-Fi and wireless networks are far below U.S. and international exposure limits for RF energy. While several studies report biological effects due to Wi-Fi-type exposures, **technical limitations prevent drawing conclusions from them about possible health risks of the technology** [(69), p. 561].	When exposure levels are maintained below current exposure limits, **neither health agencies nor guideline/standards setting organizations have identified hazards from exposure to millimeter waves or RF signals in lower frequency bands** used in previous generation technologies….while we acknowledge gaps in the scientific literature, particularly for exposures at millimeter wave frequencies, **the likelihood of yet unknown health hazards at exposure levels within current exposure limits is considered to be very low, if they exist at all** [(54), p. 244].
Simkó	In conclusion, our results demonstrate that **RF-EMF exposure** of human monocytes and lymphocytes, using different RF signals and exposure times, **does not have any activating capacity to induce ROS release** [after 30 or 45 min exposures] **or Hsp70 expression** [after 1 h exposures; (70), p. 61].	A recent careful **assessment of the scientific literature related to 5G NR transmissions does not raise alarms about possible health risks**—even as it points to limitations in the current scientific data (71) [(58), p. 724, 775].

## 3 Discussion

### 3.1 Who is speaking into the narrative and what are they saying?

The above analysis shows that:

1. The public voices expressing concern are mostly well-informed and rational, with the majority of concerns focused on safety and security.
2. Highly experienced scientists and doctors have been speaking into the 5G narrative, claiming *adequate evidence for risk of harm*. They have organized into independent science-based advocacy groups in order that their evidence-based concerns may be heard.
3. The authors claiming *no evidence of harm* are mostly industry linked and affiliated with regulatory agencies worldwide. They do not advise precaution, do not change their opinions, and they downplay the results of scientists who claim that harm exists. It seems that the strategies of big tobacco are being successfully followed by the telecommunications industry to influence RF-EMF science.

The apparent controversy comprising the 5G narrative leads to several issues and related questions, yet to be answered.

### 3.2 Maintaining doubt

Independent scientists have been stating concerns regarding health risks from all forms of wireless radiation for several decades. Now 5G has been included in those concerns. Rather than acknowledging potential harm, industry-linked author-spokespersons continue to give the impression that the science is uncertain, and harm is not confirmed. These defenders of the current standards are using an old trick, to stall new regulation by insisting that policy must be based on certainty. Such maintenance of doubt was part of the tobacco industry's disinformation campaign *long after the wider scientific community reached consensus over the health threat posed by smoking* … [(72), p. 1,036]. It is against similar obfuscation that authorities such as Hardell are speaking out (73). When two thirds of the published literature suggests biological interference and health effects from RF-EMF (64, 74), the scientific foundation for assessing the health risks of 5G is not equally weighted for and against, and the evidence is *not* inconclusive. Rather it is *suggestive of real health risks*. However, industry linked authors continue to repeat the “no conclusive evidence” mantra in order to obscure real health risks. The recent opinion piece repeats it: *... mixed results and conclusions not supporting increased risks* (5). Sound safety and protection policy is always made in the face of uncertainty and is based on a risk assessment approach (59). The question remains as to what type of evidence will industry, ICNIRP, and related scientists acknowledge as adequate evidence of harm, or as sufficient impetus to change direction.

### 3.3 Fear of the precautionary principle is unjustified

Some spokesperson-authors have claimed that precautionary messages can create undue anxiety in the public arena (75). However, a large Australian study has shown *that information about precautionary measures did not increase the risk perception of RF-EMFs during mobile phone calls* [(76), p. 1,005]. Furthermore, the results from that study suggest that risk education will not increase anxiety if it includes safety information plus incentives to use good phone hygiene practices. These results indicate that population-wide awareness and reduction of risk could coexist, and therefore, educating for precaution and risk reduction behaviors will not cause mass panic. Moreover, a precautionary approach does not need to be seen to be an impediment to economic development, because industry will find a way to implement safer technologies given the necessity.

### 3.4 The future of science and policy making

Given the current climate of maintaining doubt, and the potential positives of adopting precautionary actions, questions remain regarding the future ability of science to effectively inform policy making:

i. Why are government regulatory bodies not heeding the world's independent authorities?
ii. When will industry take responsibility for clear communication of risk?
iii. Why is the public not being educated in how to avoid potential harm?
iv. Why is the precautionary principle not being included within government policies regarding all wireless technologies, including 5G?

## 4 Concluding remarks

Consistent with professional academic integrity, experts in the field of EMF and health such as Hardell, Miller and Belyaev have been warning of harm and advocating for precaution. None of these authors have been offered ICNIRP membership or have been invited to join the WHO Environmental Health Criteria (EHC) assessment and systematic review investigation into the links between RF-EMF and a range of health outcomes (e.g., cancer, adverse reproductive outcomes, cognitive impairment, electromagnetic hypersensitivity, oxidative stress, and heat related effects).

Authors aligned with ICNIRP and/or the WHO EMF project have the ear of governments worldwide. It is this second industry-linked group who are controlling the official narrative. Members of one scientific expert group (e.g., ICNIRP) are also members of other supposedly independent expert groups (e.g., SCENIHR) (29, 34). Several of these authors have been invited to join the WHO EHC assessment and systematic review investigations, some are on more than one team, investigating very diverse discipline areas; e.g., in the teams reviewing cancer are Karipidis (humans) and Wood (animals), while Karipidis is also part of team reviewing the long-term effects on human cognition. Some of these authors have been appointed as commissioners of ICNIRP; e.g., Karipidis and more recently de Vocht (77). In this way, these researchers and the global web of advisory bodies they make up have created a stronghold that is protecting industry interests by maintaining what Maisch terms, the “Procrustean Approach” *where all scientific evidence not in conformity with the thermal bed of knowledge is simply cut off from consideration* (78). This stronghold is the foundation on which the 5G narrative and subsequent 5G policy making has been developed.

We suggest that the real problem for policymakers is that the harmful exposures that are currently being debated are created by giant global industries on which the world is becoming more and more dependent, i.e., energy and telecommunications. World dependency on any environmental toxin constitutes a “wicked problem” with uncertainty about future effects, complex interconnected issues, intractable differences in stakeholder values and resistance to change (79). Such problems need to be tackled using various strategies, including participatory and transdisciplinary processes, rational dialogue comprising public, scientific, political, and industry voices (80) and the reimagining of engineering and technology practices (79). Denigrating or silencing those scientists who are pointing out the problem is not going to help to solve it. Rather, the input of these scientists as well as the rational public is needed for the courageous problem solving that is urgently required so as to reduce RF-EMF-induced erosion of human and planetary health.

*Advocacy is less dangerous than sitting quietly on the sidelines while politicians and interest groups undermine the scientific method by perpetrating junk science*. Nature 2004 [(72), p. 1,036].

## Author contributions

SW: Data curation, Formal analysis, Investigation, Validation, Writing—original draft, Writing—review & editing. JM: Data curation, Formal analysis, Investigation, Supervision, Validation, Writing—review & editing.

## Footnotes

^1^ORSAA Database of EMF Bioeffects: https://www.orsaa.org/orsaa-database.html.

^2^Australian Communications and Media Authority.
