# Supplementary material for: Understanding the public voices and researchers speaking into the 5G narrative
Source: Front Public Health. 2024 Jan 12;11:1339513. doi: 10.3389/fpubh.2023.1339513 (PMC10820716; doi:10.3389/fpubh.2023.1339513)
Supplement: Supplementary file 2 [file Data_Sheet_2.docx]

Supplementary Material

**Supplementary document 4 - Australian authors, coauthors and their links with industry and/or military**

| **First Author** | **Coauthors** | **Co Author Industry links** |
| --- | --- | --- |
| Karipidis | Wood | Heads up a Research and Innovation Laboratory (jointly between Swinburne and Telstra).  A member of the ICNIRP SEG and has consulted with the WHO |
| Wood | Ray McKenzie | Managed the Mobile Carriers Forum (MCF), a special division of the Australian Mobile Telecommunications Association (*AMTA*) dealing with the policy communications and health and safety <https://www.linkedin.com/in/ray-mckenzie-03bb7440/?originalSubdomain=au> |
|  | Vitas Anderson | EMF consultant who has been linked to Kordia Pty Ltd a telecommunications company and Telstra Labs <https://theconversation.com/profiles/vitas-anderson-6006> |
|  | Robert L McIntosh | Associated with Telstra corporation <https://pubmed.ncbi.nlm.nih.gov/17902159/> |
|  | Vijayalaxmi | Has received funding for past work from Motorola and the US Airforce, with both organizations having a vested interest in wireless technology |
|  | Thomas Prihoda | Has co-authored many papers with Vijayalaxmi, and received funding from the telecommunications industry (Mobile Manufactures Forum and GSM Association – ODEB id 1743) and the US Airforce |

**Vijayalaxmi is well published and has received funding with cohorts for past work from Motorola and the US Airforce, with both organizations having a vested interest in wireless technology.**

| **ODEB ID** | **Date of** **Vijayalaxmi publication** | **Funding** |
| --- | --- | --- |
| 1695 | 1997 | US Airforce |
| 1712 | 2004 | US Airforce and Richard J. Fox Foundation |
| 1713 | 2001 | US Airforce and Motorola(Salary of co-author) |
| 1714 | 2003 | US Airforce and Motorola |
| 2263 | 2006 | US Airforce |
| 2264 | 2001 | US Airforce and Motorola(Salary of co-author) |
| 2265 | 2001 | US Airforce and Motorola(Salary of co-author) |
| 2266 | 2000 | US Airforce |
| 3690 | 1997 | US Airforce |
| 3691 | 1999 | US Army and US Airforce |

**Foster**

Foster - read <https://microwavenews.com/news-center/how-money-and-power-dominate-rf-research>

Foster has had at least 6 papers funded by the wireless/power industry and one funded by a government department that makes money from RF spectrum. He has made a number of comments to the editor that he has co-authored with other authors connected to industry or military or both.

**Foster papers in ODEB and funding source**

| **ODEB ID** | **Date** | **Funding source** |
| --- | --- | --- |
| 625 | 2013 | Wi-Fi Alliance and Mobile Manufacturers Forum (MMF) |
| 647 | 2013 | Electric Power Research Institute (EPRI) with technical support from Hydro One and Hydro One Brampton |
| 702 | 2012 | Collaborated with Wood and Japanese researchers for dosimetry paper - no funding declared |
| 2268 | 2019 | Collaborated with Simkó and Verschaeve - confronting bias in RF bioeffects research. Funding not declared |
| 2535 | 2017 | Collaborated with Ziskin funded by MMF |
| 3121 | 2018 | Collaborated with Ziskin and Giorgi Bit-Babik - Giorgi was a Motorola employee - Dr. Vitas Anderson provided review support; Funded by the Mobile and Wireless Forum |
| 3182 | 2018 | Collaborated with Ziskin; funded by Mobile and Wireless Forum (MWF) |
| 3936 | 2018 | Funded by the Japanese Ministry of Internal Affairs and Communications - a government body that makes money from RF spectrum licenses |
| 4357 | 2021 | Funded by Mobile and Wireless Forum (MWF) |
| 4391 | 2021 | Advised minor research funding on an unrelated topic (thermal dosimetry) from an industry group, Mobile & Wireless Forum |
| 4613 | 2023 | Collaborated with Vijayalaxmi, who has been funded in the past by the US Airforce and Motorola, and has published many “no genotoxic effect” papers (the first paper inadvertently found a significant effect that was only identified in a "correction" because of an incorrect statistical calculation in the original work – so still downplayed the outcome).The funding statement: "In the past, K. R. Foster has received minor levels of research funding from Mobile & Wireless Forum, an industry group" did not mention the GSM association or MMF and also did not mention Vijayalaxmi's own past funding from Motorola or the US Airforce. |
