# Supplementary material for: Understanding the public voices and researchers speaking into the 5G narrative
Source: Front Public Health. 2024 Jan 12;11:1339513. doi: 10.3389/fpubh.2023.1339513 (PMC10820716; doi:10.3389/fpubh.2023.1339513)
Supplement: Supplementary file 3 [file Data_Sheet_3.DOCX]

Supplementary Material

**Supplementary Document - 3 Minutes from ARPANSA meetings 2018-2022 showing the relationships between ARPANSA, ICNIRP, Australian research institutions, ACMA and the WHO.**

Sourced from Aust Govt Transparency Portal ARPANSA annual report, Appendix 2:

**Operations of the Radiation Health and Safety Advisory Council and Committees**

<https://www.transparency.gov.au/annual-reports/australian-radiation-protection-and-nuclear-safety-agency/reporting-year/2021-22-29>

Click on [www.arpansa.gov.au/rhsac-minutes](https://www.arpansa.gov.au/about-us/advisory-council-and-committees/radiation-health-and-safety-advisory-council/minutes).

- … *ARPANSA’s input on non-ionising radiation to the World Health Organization (WHO) demonstrating Australia’s influence in developing international standards and strategies for risk reduction.*

Radiation Health and Safety Advisory Council 1-2 August 2019 FINAL Minutes <https://www.arpansa.gov.au/sites/default/files/final_council_minutes_1-2_august_2019.pdf>

- *Also highlighted were … attendance at an International Commission on Non-Ionising* *Radiation Protection (ICNIRP) meeting in Paris*

Radiation Health and Safety Advisory Council 5-6 March 2019 Final Minutes <https://www.arpansa.gov.au/sites/default/files/final_council_minutes_5-6_march_2019.pdf>

- *Item 2.2 Electromagnetic energy (EME) funding*

*Council discussed the need for ARPANSA to have a role in EME research and advice to the public, particularly with regard to the rollout of 5G technology. Council was provided with background on the status of government funding allocated to ARPANSA for an EME program, which has remained static. A key concern to ARPANSA is the diminishing capability for investment into* ***EME Centres of Research Excellence, which draw together researchers and universities in this specialised research field, to feed into an international research agenda that informs the international standards upon which ARPANSA depends, as a basis for advice given to the Australian public.***

*It was noted that ARPANSA has initiated discussions with relevant government stakeholders including Department of Communications and the Arts (DoCA), the Australian Communications and Media Authority (ACMA), the National Health and Medical Research Council (NHMRC), and Department of Health, to address the long-term sustainability of the EME Program.*

*Action: Council to send a letter to the CEO of ARPANSA in support of continued funding from Department of Communications and the Arts for the EME Program.*

Radiation Health and Safety Advisory Council 6-7 March 2018 Draft Minutes <https://www.arpansa.gov.au/sites/default/files/rhsac-minutes-6-7-march-2018.pdf>

*Item 2.1 Precautionary approach to IR, UVR and EMR (working group 3)*

*A paper was presented on the current state of progress towards* ***guidance around the low dose effects of ultraviolet and electromagnetic radiation (UVR/EMR)****. It was noted that ARPANSA can bring further developments on UVR/EMR advice to Council for consideration at the relevant time in the future*

*Item 5.1 Radiation risk management*

*Council was provided with a* ***proposed approach to the management and communication of health and environmental risk****, including a visual tool to improve public messaging about the risks of different types of radiation. The proposal was endorsed as a significant improvement over existing messaging, and Council strongly endorsed the proposal for further exploration and development.*

<https://www.arpansa.gov.au/sites/default/files/rhsac_-_council_minutes_16-17_november_2017_final.pdf>

Radiation Health and Safety Advisory Council 16-17 November 2017 Draft Minutes

*Item 3.5 News media perceptions of radiation and ARPANSA Niall Byrne*

*RHSAC held a question and answer forum with invited journalists to* ***discuss how ARPANSA and radiation is perceived in the media****. Members discussed opportunities for* ***ARPANSA to enhance its media messaging*** *and the importance of ARPANSA providing a credible voice in its scientific role wherever interest groups dominate public discourse.* ***The importance of maintaining trusted relationships with media was noted****. Members discussed the broad context of ARPANSA’s role to provide information on radiation risks in society, and* ***how media may influence behavioural change****. The advantages of* ***involving the media early in a public consultation process*** *were also discussed.*

Radiation Health and Safety Advisory Council 5-6 June 2017 Draft Minutes

# <https://www.arpansa.gov.au/sites/default/files/mins-jun17.pdf>
