# Supplementary material for: Understanding the public voices and researchers speaking into the 5G narrative
Source: Front Public Health. 2024 Jan 12;11:1339513. doi: 10.3389/fpubh.2023.1339513 (PMC10820716; doi:10.3389/fpubh.2023.1339513)
Supplement: Supplementary file 4 [file Data_Sheet_4.DOCX]

**Supplementary document 1: Full profiles of author-spokespersons contributing to the 5G narrative**

We investigated the full profiles of the authors critiqued in the opinion piece, as well as three important 5G-review paper authors who were omitted (see below). Each author’s publications pertaining to health effects from wireless electromagnetic fields were extracted from ODEB ^1,2^ In addition, their clinical or research experience and their additional works were investigated using Pubmed ^3^, Google Scholar, ResearchGate and institutional websites.

Each author was first classified according to whether they come from a clinical, radiological or scientific research background and how much experience they have had in their own field. Each was also classified according to type of experience they have had in the field of RF-EMF and health; i.e. an advocate speaking from a position of first-hand scientific or clinical experience, a researcher who uses the works of others to build new theory or methodologies, or an analyst who investigates the work of others and makes reasoned arguments from this analysis. For each of these types the level of experience is also noted (highly experienced, experienced, or novice).

| **Researcher**  **(Classification by de Vocht and Albers)** | **Profile of author’s expertise** | **Expertise in own field** | **Expertise in health effects of RF-EMF** |
| --- | --- | --- | --- |
| Di Ciaula, A.  **(Activism)** | - Clinical medicine researcher in Biomedical Sciences and Human Oncology, University of Bari, Italy. - Mostly experimental work in the area of gallbladder disease, with 116 papers listed in PubMed. - Listed in ODEB: two full systematic reviews, one on thyroid disease and environmental toxins and the other a review of biological effects of millimeter waves (a high-quality systematic review). - A member of the International Society of Doctors for Environment (ISDE). | Highly Experienced Medical Researcher | Analyst |
| Russell, CL.  **(Activism)** | - Medical doctor/plastic surgeon. - Treated breast cancer patients over 3 decades. - Chair of the Santa Clara County Medical Association (SCCMA/MCMS) Environmental Health Committee. - Executive Director of Physicians for Safe Technology^4^ - Advocate and writer for policy changes to reduce environmental toxins (e.g., pesticides). - Listed in ODEB: 2 review papers: one detailing the health risks of RF-EMF on health, the other a proposed set of building codes for EMF-safe buildings. | Highly Experienced Clinician | Experienced Analyst |
| McClelland, S 3rd  **(Independent)** | - Senior Clinical radiation oncologist in Radiation Medicine at Oregon Health and Science University, including university hospital work with glioblastoma patients. - Co-author of a paper looking at RF fields (200kHz) for Brain tumor treatment - Over 200 publications listed on Google Scholar late 1980s to present; one paper (letter) in ODEB. - A letter to the editor in an oncology journal - based on evidence of harm to hippocampal cells and human neuroblastoma cells from wireless RF-EMF, McClelland et al. asked whether clinicians *with understanding of radiation safety* should ignore the issue or respond in some way. | Highly Experienced Medical Researcher and Clinician | Highly Experienced in Ionizing Radiation and Experienced in Non-Ionizing Radiation |
| Miller, A.B.*  **(Activism)** | - Professor Emeritus / Epidemiologist in Public Health (oncology) at the University of Toronto since the early 90’s - 656 papers listed on ResearchGate <https://www.researchgate.net/profile/Anthony-Miller-3> - Listed in ODEB: 4 case control studies (occupational / residential exposures to ELFs / MFs and cancer / leukemia), 2 papers on the development of indices for measuring ELFs and MFs; 5 reviews, 2 opinion pieces, 1 comment paper. - Began cautious about effects, changed opinion based on results from research, now advocating for greater protection. - A Former Advisor to the World Health Organization and senior epidemiologist for IARC. - Has served as plaintiff’s expert witness in cases involving radiofrequency radiation. | Highly Experienced Epidemiologist | Highly Experienced |
| Hardell, L.*  **(Activism)** | - Medical Doctor/ Oncologist, Researcher in Medicine and Health, Sweden for over 3 decades, now retired. - Many epidemiology case control studies on environmental toxins and cancer (over 100 research papers)   - 1990s-2000s PCBs, dioxins organic solvents and cancer   - 2000 – 2023 EMF and cancer - A member of the 2011 IARC committee investigating the carcinogenicity of radio/microwave frequencies ^5^ - Studies in ODEB include 69 research papers (33 Effect, 5 no effect) and 31 review / opinion papers. - Runs the Environment and Cancer Foundation^6^ to fund his research. | Highly Experienced Oncologist and Epidemiologist | Highly Experienced |
| Kostoff, R.N.  **(Independent /Activism)** | - Researcher in Public Policy at Georgia Institute of Technology from Late 1990s onwards. - Data mining, use of metrics in assessing quality of research and setting public exposure limits and synergistic effects of multiple toxins with adverse effects of EMFs. - 93 papers listed on ResearchGate. - Listed in ODEB: adverse effects of EMF in conjunction with other biotoxins: 2 review monographs, recommendations for determining exposure metrics: 3 opinion papers and 1 editorial. | Highly Experienced Researcher | Experienced Researcher - Analyst |
| Bushberg, J.T.  **(Industry)** | - Professor of Radiology Physics at University of California, background in ionizing radiation safety in medical settings. - 53 papers listed on ResearchGate. - Listed in ODEB: 2 papers - Co-author of the IEEE standards for wireless radiation. - Vice-Chair of COMAR, Technical Committee of IEEE EMBS. - Worked in the military and advised governments and regulatory bodies (FDA, WHO) ^7^. - Co-author on this paper is Kenneth Foster. | Experienced Radiological Health Physicist | Highly Experienced (Industry-linked) |
| Foster, K.R.  **(New)** | - 4 decades in the research of dielectrics in medicine (over 400 references listed in google scholar from the 80s onwards). - Foster has only 1 experimental paper in ODEB, the other 22 papers are theoretical modelling, reviews or opinions and 7 of these papers acknowledge industry or military funding. - Foster dismisses non-thermal effects and suggested mechanisms. - Foster was a student and colleague of Herman Schwan, a German scientist who was recruited by the US military after the war. Schwan is seen as the father of biomedicine. However, Schwan’s theorizing and modelling was from the perspective of physical and macro biophysics, which served him well in developing diathermy in medicine. However, Schwan did not move into the developing fields of biology or quantum perspectives as they became better understood. Thus, Schwan was unable to understand how non-thermal effects could exist. The Schwan-led 1966 IEEE committee set the first US wireless exposure limit of 10mW/cm2. - Foster has followed in the footsteps of his mentor, reiterating the “thermal only” theory and dismissing any alternative theories for 4 decades. | Highly Experienced Industry linked Physicist (theoretical modelling) | Physicist (Theoretical modelling) |
| Leszczynski, D.*  **(Independent)** | - Adjunct Professor at the University of Helsinki and has a background in Biochemistry, Cell biology and Radiation Proteomics. - 120 papers listed on ResearchGate. Papers listed in ODEB: 11 experimental (8 effects, 3 no effect, 1 uncertain effect), 4 review/opinion papers and 1 methodology proposal. - A member of the 2011 IARC committee investigating the carcinogenicity of radio/microwave frequencies ^5^ - Specialty Chief Editor of Frontiers in Public Health (Radiation and Health). - Runs a blog aimed at critiquing scientific quality of papers on both sides of the debate. | Experienced Scientist and Researcher | Experienced Scientist and Highly Experienced Analyst |
| Frank, J.W.  **(Activism)** | - Epidemiologist at the Uni of Edinburgh in many areas of pop health, public health and health policy. - 219 papers listed in PubMed. - Listed in ODEB: One essay on RF-EMF and public policy. | Highly Experienced Epidemiologist | Analyst |
| Karipidis, K.K.  **(Independent)** | - Health Physicist in application of non-ionising radiation since 2000 at the Australian Radiation Protection and Nuclear Safety Agency’s (ARPANSA). - Epidemiology PhD completed in 2017 - Health Impact Assessment Assistant Director and Associate Professor at ARPANSA, a government radiation protection agency. - Member of ICNIRP project groups (review of intended human exposure to non-ionising radiation for cosmetic purpose) ^8^. - Listings in Pubmed: 23 papers on ultraviolet exposure, sunscreen and exposures to microwaves - Listed in ODEB: 4 epidemiology studies: 2 case control (no effect) and 2 ecological (uncertain effects); 2 dosimetry field studies – one measuring magnetic fields from power lines, one ‘school exposure’ study (measuring 6-minutes of RF exposures emitted by one laptop in empty classrooms), 1 commentary, 1 feasibility study, 1 metanalysis, 3 systematic review/map protocols, 1 systematic map (effects on the environment), 2 reviews, 2 letters to the editor - 8 papers are co-authored with Croft or Wood or Feychting, who are industry linked/funded. | Experienced Health Physicist (UV radiation and cosmetics) and recent Epidemiologist | Analyst  (Government employed, Industry linked) |
| Wood, A.W.  **(Independent)** | - Chief Investigator at Swinburne University of Technology. - Has been member of the ICNIRP Scientific Expert Group (SEG) from 2013 until 2021 and collaborates with the Australian Radiation Protection and Nuclear Safety Agency ^9^. - 56 papers listed in PubMed. - Listed on ResearchGate: 122 papers since 1972, with a focus on mathematical / computational modelling of biophysical processes related to EMF exposures. - Listed in ODEB: 13 experimental papers (6 effect, 6 no effect, 1 uncertain effect); 3 modelling papers, 3 reviews, 1 meta-analysis, 5 opinion/commentaries, 3 systematic review protocols, 1 systematic map - Of these, there are 15 papers that are funded or receive technical support from the telecommunications industry, including 8 papers with co-authors who have received a salary from industry. - Collaborative partnership with telecommunications Industry (Telstra) supported and funded ‘innovation lab’ at Swinburne University ^10^. - Previously directed a research group which included two technical associates who are employed by the telecommunications industry. - Has co-authored with Foster. | Highly experienced Industry linked Engineer (theoretical modelling) | Highly experienced |
| Jargin, S.V.  **(Independent)** | - Medical Doctor/ Pathologist. - Peoples' Friendship University of Russia Specializing in Hypercholesterolemia, Lipid Metabolism. - 698 articles in ResearchGate since 1986, most are opinion papers. - Several opinion papers dismissing effects of environmental toxins such as “Overestimation of cardiovascular consequences of low dose low-rate ionizing radiation” 2023 and “Asbestos-related Cancer: Exaggerated Risk Perception” 2023 | Highly experienced medical doctor | Novice  (Industry supporter) |
| Lin, J.  **(omitted)** | - Professor Emeritus with the University of Illinois. - 177 publications listed on IEEE explore since 1973 ^11^. - Research has focused on electromagnetics in biology and medicine including invention of medical applications. - Previous positions held:   - President of the Bioelectromagnetics Society   - Chairman for the International Scientific Radio Union   - Commission on Electromagnetics in Biology and Medicine   - Vice President for the U.S. National Council on Radiation Protection and Measurements (NCRP)   - Past Member of the International Commission on Nonionizing Radiation Protection (ICNIRP) - Listed in American Men and Women of Science, Who’s Who in America, Who’s Who in Engineering, Who’s Who in the World, and Men of Achievement. | Highly Experienced Radiological Engineer | Highly Experienced |
| Belyaev, I.Y.*  **(omitted)**  PhD Radiobiology (biophysics) | - Head of Department at Biomedical Research Centre Slovak Academy of Sciences. - Experimental research in genetics, biophysics, and cancer since 1986. - A member of the 2011 IARC committee investigating the carcinogenicity of radio/microwave frequencies ^5^. - Commissioner of the ICBE-EMF. - 148 articles listed on ResearchGate since 2006. - First review of biological effects of millimeter waves in 1992. - First author on a 2019 IEEE review paper on millimeter waves ^12^. - First author of *EUROPAEM EMF Guideline 2016 for the prevention, diagnosis and treatment of EMF-related health problems and illnesses.* - Listed in ODEB: 28 Experimental works since early 1990s mostly on effects of mmw on DNA and E-coli all showing effects; 3 theoretical papers on and mechanisms of action of microwaves on human cells; exposure modelling; 10 opinion /review papers. - Awarded “most influential paper in Bioelectromagnetics 2006-2010” by the Bioelectromagnetics Society in 2011. - Has served as plaintiff’s expert witness in cases involving radiofrequency radiation. - Development of theoretical understanding can be seen over 3 decades. | Highly Experienced Biophysicist | Highly Experienced |
| Nizhelska, O.I.  **(Omitted)** | - Russian and Ukrainian research review 2020 ^13^. - Russian biophysicist expert in effects of electromagnetic fields on yeast and bacterial cells – in biological and industry applications. - Listed in ODEB: 2 experimental studies (effects found), 3 review/opinion papers | Highly Experienced Biophysicist | Experienced |
| Belpoggi, F. *  **(New)** | - Experimental work researching Cancer and Toxicology in Animals since 2001 (over 20 years). - 115 articles listed on ResearchGate. - Research Interest Score higher than 97% of ResearchGate members. - Fellow of the International Academy of Toxicologic Pathology Fellow (IATPF). - Former Research Director of L'Istituto Ramazzini (Ramazzini Institute, Bologna, Italy), advisor since 2019 ^14^. - Director of the Ramazzini Institute’s long-term RF exposure study^15^, which found schwannoma of the heart among exposed rats, leading to the call for IARC review of existing classification of RF as a cancer-causing agent ^16^. - Author of the 2021 European Parliament commissioned Panel for the Future of Science and Technology report Health Impact of 5G ^17^. - Listed in ODEB: 2 experimental studies with rats (effects found), 2 review/opinion papers, 1 letter to the editor | Highly Experienced Scientist (Biology- Toxicology) | Highly Experienced |
| Simko, M.  **(Industry)** | - Experimental work researching cellular and biochemical changes from low frequency (50Hz) electric and magnetic fields (most papers show effects). Only 4 papers in the area of RF frequencies: two that showed no effects and two from the same project that showed uncertain effects. - 20 papers listed in ODEB from 2001 to 2021. - Has coauthored with Foster. - Funded by the German Federal Office for Radiation Protection and Deutsche Telekom. | Highly Experienced Biophysicist | Experienced  (Industry Linked) |
| *These authors advocated in 2016 for the European Food Safety Authority (EFSA) to recognize glyphosate as a probable human carcinogen, by using IARC principles to critique the EFSA position ^18^ | | | |

References

1. Leach V, Weller S, Redmayne M. A novel database of bio-effects from non-ionizing radiation. *Reviews on environmental health*. 2018;33(3):273-280. <https://www.degruyter.com/document/doi/10.1515/reveh-2018-0017/html>

2. Leach V, Weller S, Redmayne M. Authors' Reply to Drießen's Letter to the Editor on "A novel database of bio-effects from non-ionizing radiation" *Reviews on environmental health*. 2019;34(1):101-103. doi:10.1515/reveh-2018-0077. <https://www.ncbi.nlm.nih.gov/pubmed/30710488>

3. National Library of Medicine National Centre for Biotechnology Information. Pubmed. <https://pubmed.ncbi.nlm.nih.gov/>

4. Physicians for Safe Technology. Cindy Russell, M.D. 2016;<https://mdsafetech.org/advisory-board/cindy-russellmd/>

5. IARC Working Group on the Evaluation of Carcinogenic Risks to Humans. *Non-ionizing radiation, Part II: Radiofrequency electromagnetic fields*. IARC monographs on the evaluation of carcinogenic risks to humans. International Agency for Research on Cancer,; 2011. <https://www.researchgate.net/publication/236332981_Non-ionizing_radiation_Part_II_Radiofrequency_electromagnetic_fields_IARC_Working_Group_on_the_Evaluation_of_Carcinogenic_Risks_to_Humans_2011_Lyon_France>

6. Environment and Cancer Research Foundation. <https://environmentandcancer.com/>

7. IEEE Xplore. Jerrold Bushberg Biography. <https://ieeexplore.ieee.org/author/37088825720>

8. Australian Radiation Protection and Nuclear Safety Agency (ARPANSA). ARPANSA scientist Dr Ken Karipidis joins international commission. 2021; <https://www.arpansa.gov.au/news/arpansa-scientist-dr-ken-karipidis-joins-international-commission>

9. International Commission on Non-Ionizing Radiation Protection. Declaration of personal interests. <https://www.icnirp.org/cms/upload/doc/WoodDOI2019.pdf>

10. Swinburne University. 6G Research and Innovation Laboratory. 2023;<https://www.swinburne.edu.au/research/facilities-equipment/6g-research-innovation-laboratory/>

11. IEEE Xplore. James C. Lin Biography. <https://ieeexplore.ieee.org/author/37278769800>

12. Belyaev I. Main Regularities and Health Risks from Exposure to Non-Thermal Microwaves of Mobile Communication. presented at: 14th International Conference on Advanced Technologies, Systems and Services in Telecommunications (TELSIKS); 2019; Nis, Serbia.

13. Lantow M, Lupke M, Frahm J, Mattsson M, Kuster N, Simko M. ROS release and Hsp70 expression after exposure to 1,800 MHz radiofrequency electromagnetic fields in primary human monocytes and lymphocytes. *Radiation and environmental biophysics*. 2006;45:55-62.

14. Collegium Ramazzini. Belpoggi Fiorella: Ramazzini Award recipient and Ramazzini Lecturer for 2007. <https://www.collegiumramazzini.org/download/2007_CERTIFICATO_ONOREM_Belpoggi.pdf>

15. Falcioni L, Bua L, Tibaldi E, et al. Report of final results regarding brain and heart tumors in Sprague-Dawley rats exposed from prenatal life until natural death to mobile phone radiofrequency field representative of a 1.8 GHz GSM base station environmental emission. *Environmental research*. 2018;165:496-503. <https://www.sciencedirect.com/science/article/abs/pii/S0013935118300367>

16. Italian RF–Animal Study: “Consistent with” and “Reinforces” U.S. NTP Cancer Finding. Ramazzini’s Belpoggi Calls for IARC To Reassess RF–Cancer Risk. <https://microwavenews.com/news-center/ramazzinis-belpoggi-interview>.

17. European Parliamentary Research Service report: Health impact of 5G, Study for the Panel for the Future of Science and Technology (European Parliamentary Research Service, Scientific Foresight Unit,) (2021). <https://www.europarl.europa.eu/RegData/etudes/STUD/2021/690012/EPRS_STU(2021)690012_EN.pdf>

18. Portier CJ, Armstrong BK, Baguley BC, et al. Differences in the carcinogenic evaluation of glyphosate between the International Agency for Research on Cancer (IARC) and the European Food Safety Authority (EFSA). *J Epidemiol Community Health*. 2016;70(8):741-745.
